# The cytokine expression in synovial membrane and the relationship with pain and pathological findings at hip arthroscopy

**DOI:** 10.1186/s40634-017-0086-4

**Published:** 2017-04-20

**Authors:** Kensuke Fukushima, Gen Inoue, Hisako Fujimaki, Kentaro Uchida, Masayuki Miyagi, Naoshige Nagura, Katsufumi Uchiyama, Naonobu Takahira, Masashi Takaso

**Affiliations:** Department of Orthopaedic Surgery, Kitasato University School of Medicine, 1-15-1, Kitazato, Minami-ku, Sagamihara, 252-0374 Kanagawa Japan

**Keywords:** Hip arthroscopy, Hip pain, Synovial membrane inflammation, Labral tear, Cytokine

## Abstract

**Background:**

Synovial membrane inflammation is the most common finding presenting during hip arthroscopy, and may play a role in hip pain. We sought to determine the relationships between synovial cytokine levels, hip pain, and arthroscopic findings of the hip joint.

**Methods:**

We prospectively included 33 patients who underwent arthroscopic hip surgery (34 hips). For all patients, radiographs and severity of pain were evaluated preoperatively. During arthroscopy, we classified the chondral injury and synovitis, noted the incidence of labral tear and its instability, and a sample of the synovial membrane was harvested for quantitative PCR to determine levels of TNFα, IL1β, IL6, ADAMTS4, MMP1, and MMP3. The relationships between the levels of these cytokines, severity of hip pain, and the pathological findings during arthroscopy were examined.

**Results:**

Pain intensity and cytokine levels were not significantly different between patients with labral tear or instability and those without. By contrast, the expression of TNFα, IL1β, IL6, and MMP1 mRNA was significantly higher in patients with diffuse synovitis than in patients with focal synovitis. VAS score during rest showed significant positive correlation with IL6 (*r* = 0.45, *p* < 0.01), while VAS score on walking showed a positive correlation with TNFα (*r* = 0.47, *p* < 0.01), and ADAMTS4 (*r* = 0.51, *p* < 0.01). The modified Harris Hip pain score showed a negative correlation with TNFα (*r* = −0.38, *p* = 0.04) and IL6 (*r* = −0.58, *p* < 0.01).

**Conclusions:**

The severity of synovitis and chondral injury are considered to be more important in the pathology of hip pain than labral tear or instability. Inflammatory cytokines, especially TNFα and IL6 might play an important role in the pathogenesis of pain in patients indicated for hip arthroscopy, possibly depending on the severity of synovitis.

## Background

Hip arthroscopy has become a common technique for both the diagnosis and treatment of painful hips (Bedi et al. 2013). The main objectives of arthroscopic hip treatment are treatment of labral tears and chondral injuries, which are generally considered to be sources of pain and disability (Bedi et al. 2013; Beaule et al. 2009). Although treatment of labral tears by hip arthroscopy has been shown to decrease pain and improve function, a subset of patients have persistent pain, and the origin of that pain cannot be accounted for completely (Byrd and Jones 2009). By contrast, a cross-sectional survey of asymptomatic adolescents who underwent MRI revealed labral tears in 13% (19/80) of female patients and in 34% (34/244) of male patients, which means the labral tear itself sometimes occurs without pain (Nelson 2014).

Synovial membrane inflammation is the most commonly presenting finding during hip arthroscopy, and may have a role in the pain state. Synovial hyperplasia is present in patients with knee pain and osteoarthritis (OA) more often than in people with OA, but without pain (Scanzello et al. 2013). We considered that a reason for increased pain in some patients may be the presence of inflammatory cytokines within the synovial membrane, which may stimulate nociceptors and signify pathological processes in the hip joint, as is reported for the knee joint (Bellucci et al. 2013; Higuchi et al. 2006; Orita et al. 2011). However, there have been few studies concerning cytokine levels in the hip joint. To our knowledge, there have been no studies to assess the correlation between cytokine level and hip pain. Importantly, the pathological features that can be detected during hip arthroscopy (i.e. synovium inflammation, labral tears, and chondral injuries) inducing hip joint pain have not yet been fully elucidated.

Therefore, we aimed to determine relationships between synovial cytokine levels, hip pain, and pathological findings during hip arthroscopy, to clarify the pathomechanism of hip pain in patients who have undergone arthroscopy.

## Methods

### Patients

We obtained approval for this study from the Institutional Review Board for Clinical Research and Treatment in Kitasato University (approval No. B13-113), and the study was performed in accordance with the ethical standards laid down in the 1964 Declaration of Helsinki and its later amendments. All patients included provided their written informed consent to participate in the present study. We prospectively included 33 patients who were about to undergo arthroscopic hip surgery (34 hips). Exclusion criteria were patients aged 60 years or older, patients diagnosed with OA severity of Tönnis grade 3 or higher, or with inflammatory disease such as rheumatoid arthritis and purulent arthritis, or synovial chondromatosis. All patients complained of groin pain, restricted hip motion on flexion and internal rotation, and had a positive anterior impingement test. Our indications for arthroscopic hip surgery were labral tears and/or increased synovial fluid identified with pre-operative magnetic resonance imaging (MRI), and pain relief with 10 mL injection of 0.5% procaine hydrochloride into the hip joint at the initial visit. All patients were resistant to conservative treatment with medication and physical therapy, which was indicated for at least 3 months. During these 3 months, no patients were medicated with non-steroidal anti-inflammatory drugs or steroids. For all patients, age, sex, and clinical history were recorded. Physical examination, antero-posterior (AP) and cross-lateral plain radiographs, and MRI were evaluated. The pain component of the modified Harris Hip Score (mHHS) and visual analog scale (VAS) score at rest and on walking were evaluated preoperatively (Murray 1993).

The centre-edge (CE) angle of the hip joint was measured using an AP plain radiograph. AP plain radiographs of the hip joint were assessed for the presence of coxa profunda and protrusio acetabuli, and CE angle ≥40°, which are indicators of a deep socket, a crossover sign known as an indicator of acetabular retroversion. The femoral α angle was measured; hip joints with a femoral α angle ≥55° were defined as having cam deformity. Patients having hip joints with a deep socket, acetabular retroversion or femoral cam deformity, or both, were defined as having femoroacetabular impingement (FAI) (Tannast and Siebenrock 2009). OA grade was assessed radiographically according to the Tönnis classification system (Tonnis 1987).

### Arthroscopic assessment

For hip arthroscopy, patients were placed in the supine position upon a fracture table. Two portals (antero-lateral and mid-anterior) were used in all cases. Gentle traction was applied to bilateral legs and a spinal needle was used to establish the anterolateral portal over a guide wire using fluoroscopy. With the camera in the anterolateral portal, the spinal needle was introduced into the location of the mid-anterior portal. By way of these two portals, we assessed the incidence of any labral tear and its instability as reported by Lage et al., and classified any chondral injury using the Outerbridge classification (Lage et al. 1996; Outerbridge 1961). In addition, we assessed synovitis (inflammation of the synovial membrane) and classified it as follows: grade 1, no synovitis; grade 2, focal synovitis at the labral tear; grade 3, diffuse synovitis in the area surrounding the joint. We harvested a sample of the synovial membrane from the site of strongest synovitis for further evaluation when we performed the capsulectomy. For instability of the labrum, which was classified as a peripheral longitudinal labral tear using Lage’s classification, we performed labral fixation using anchors (Lage et al. 1996). All operations were performed by a single surgeon. The classification of labral tears, instability, chondral injury, and synovial membrane inflammation was made in collaboration with two experienced hip surgeons.

### Isolation of RNA and quantitative RT-PCR

Total RNA was extracted from harvested synovial samples using TRIzol (Invitrogen, Carlsbad, CA) according to the manufacturer’s instructions, and was used as a template for first-strand cDNA synthesis using SuperScript III RT (Invitrogen). The PCR reaction mixture consisted of 2 μL cDNA, the specific primer set (0.2 μM final concentration), and 12.5 μL SYBR Premix Ex Taq (TaKaRa, Kyoto, Japan) in a final volume of 25 μL. The sequences of the PCR primer pairs are listed in Table 1. Quantitative PCR was performed using a real-time PCR detection system (CFX-96; Bio-Rad, Hercules, CA). The PCR cycle parameters consisted of an initial denaturation at 95 °C for 1 min, followed by 40 cycles of 95 °C for 5 s, and 60 °C for 30 s. mRNA expression was normalized to the level of GAPDH mRNA.

**Table 1:** Sequences of the primers used in this study

Gene	Direction	Primer Sequence (5’–3’)	Product Size (bp)
TNFα	F	CTTCTGCCTGCTGCACTTTG	118
	R	GTCACTCGGGGTTCGAGAAG	
IL1β	F	GTACCTGTCCTGCGTGTTGA	153
	R	GGGAACTGGGCAGACTCAAA	
IL6	F	GAGGAGACTTGCCTGGTGAAA	199
	R	TGGCATTTGTGGTTGGGTCA	
ADAMTS4	F	ACGTGCTCCATGACAACTCC	159
	R	TGCCCATAGCCATTGTCCAG	
MMP1	F	ACTTACATCGTGTTGCGGCT	164
	R	CGATGGGCTGGACAGGATTT	
MMP3	F	GTGGAGTTCCTGACGTTGGT	164
	R	TGGAGTCACCTCTTCCCAGA	
GAPDH	F	TGTTGCCATCAATGACCCCTT	202
	R	CTCCACGACGTACTCAGCG	

Six molecular biomarkers of inflammation within the synovium were measured: tumor necrosis factor-α (TNFα), interleukin-1β (IL1β), IL6, A disintegrin and metalloproteinase with thrombospondin Motifs-4 (ADAMTS4), matrix metalloproteinase-1 (MMP1), and MMP3.

We determined the correlations between VAS score or mHHS pain score and arthroscopic findings. The relationships between pain intensity or arthroscopic findings and cytokine levels in the synovial membrane were determined.

### Statistical analyses

Statistical analyses were performed using JMP version 11.0 software (SAS Institute, Cary, NC). Continuous variables are expressed as the mean and the standard error of the mean unless otherwise indicated, while categorical variables are expressed as *n* (%). A Pearson chi-squared test was used to determine the correlations between labral tear or labral instability and inflammation of the synovial membrane. A non-parametric Mann-Whitney *U* test was used for comparisons of normally distributed data among the groups (labral tear or instability, Outerbridge classification, and classification of synovitis). When the minimum detectable difference was considered as 2.5 for VAS on walking, with 80% power and an α level of 0.05 the sample size was calculated as 24. A *p* < 0.05 was considered significant. Correlation coefficients were analysed to assess the associations between VAS, pain score of mHHS, and synovial cytokine levels.

## Results

In 33 patients (34 hips), there were 10 men (11 hips) and 23 women (23 hips) with an average age of 41.8 ± 2.5 years. Their mean pre-operative VAS score was 27.3 ± 3.2 during rest and 59.4 ± 3.5 on walking. Their pre-operative mean mHHS score was 57.5 ± 1.8 in total and the pain component was 25.6 ± 1.1. In plain radiographs, 22 hips (64.7%) were classified as Tönnis grade 0, 10 hips (29.4%) as grade 1, and 2 hips (5.88%) as grade 2. The radiographic findings related to FAI in 16 hips (47.1%) were investigated.

Based on arthroscopic findings, 29 hips (85.3%) were identified as having labral tears. Of the 29 hips with labral tear, labral instability was identified in 18 hips (62.1%), which needed labral fixation. In the Outerbridge classification, 5 hips (14.7%) were grade 0 (normal cartilage), 16 hips (47.1%) were grade 1 (cartilage with softening and swelling), 7 hips (20.6%) were grade 2 (partial-thickness defect with fissures on the surface that do not reach subchondral bone or exceed 1.5 cm in diameter), 4 hips (11.8%) were grade 3 (fissuring to the level of the subchondral bone in an area with a diameter more than 1.5 cm), and 2 hips (5.9%) were grade 4 (exposed subchondral bone). No hip was identified as grade 1 (no synovitis) by our classification, 16 hips (47.1%) as grade 2 (focal synovitis), and 18 hips (52.9%) as grade 3 (diffuse synovitis). We found no significant correlations between labral tear or labral instability and inflammation of the synovial membrane.

### Comparison of pain severity between patients classified with various arthroscopic findings

Based on the arthroscopic findings, none of the VAS scores during rest, VAS scores on walking, or mHHS pain score were significantly different between patients with or without labral tear or instability, or between patients with focal or diffuse synovitis (Table 2). The severity of chondral injury classified by Outerbridge grading tended to show increased VAS score during rest and on walking, and decreased mHHS pain score, but the differences found were not significant. Compared with patients with grade 2 (focal) synovitis, patients with grade 3 (diffuse) synovitis tended to have a higher VAS score on walking and lower mHHS pain score, but the differences were not significant.

**Table 2:** Comparison of pain severity in patients classified by arthroscopic findings

	VAS on rest	VAS on walking	mHHS	Pain score of mHHS
Labral tear				
+ (*n* = 29)	27.6 ± 3.6	59.7 ± 3.9	58.7 ± 2.5	25.5 ± 1.2
– (*n* = 5)	26.0 ± 8.6	58.0 ± 9.4	56.9 ± 11.1	26.0 ± 2.8
Labral instability				
+ (*n* = 18)	29.4 ± 4.5	58.3 ± 4.9	59.0 ± 4.0	25.6 ± 1.5
– (*n* = 16)	25.0 ± 4.8	60.7 ± 5.2	57.8 ± 4.1	25.6 ± 1.6
Chondral injury				
0 (*n* = 5)	33.0 ± 8.6	62.0 ± 9.1	51.7 ± 13.8	24.0 ± 2.8
I (*n* = 16)	21.3 ± 4.8	53.5 ± 5.1	63.9 ± 1.8	27.5 ± 1.5
II (*n* = 7)	31.1 ± 7.2	57.7 ± 7.7	57.2 ± 6.6	24.3 ± 2.3
III (*n* = 4)	30.0 ± 9.6	75.0 ± 10.2	56.1 ± 4.0	25.0 ± 3.1
IV (*n* = 2)	42.5 ± 13.6	75.0 ± 14.4	50.6 ± 16.5	20.0 ± 4.3
Synovitis				
2: focal (*n* = 16)	26.6 ± 4.8	56.4 ± 5.2	62.7 ± 1.7	27.5 ± 1.5
3: diffuse (*n* = 18)	28.0 ± 4.6	62.1 ± 4.9	52.3 ± 5.8	23.9 ± 1.4

^*^ *P* < 0.05 was considered significant

### Comparison of cytokine levels between patients with various pathological findings during hip arthroscopy

We compared cytokine levels with pathological findings during hip arthroscopy (Table 3). Existence of labral instability was not statistically correlated with any cytokine level. However, in patients with chondral injury classified as Outerbridge grade 4, the levels of TNFα were significantly increased. In patients with grade 3 cases of synovitis, the levels of TNFα, IL1β, IL6, and MMP1 were significantly increased compared with those in cases of grade 2 synovitis (*p* < 0.05).

**Table 3:** Comparison of cytokine levels in patients classified by pathological findings during hip arthroscopy

	TNFα	IL1β	IL6	ADAMTS4	MMP1	MMP3
Labral tear						
+ (*n* = 29)	0.23 ± 0.2	0.016 ± 0.006	0.0082 ± 0.006	0.074 ± 0.03	0.49 ± 0.2	1.7 ± 0.9
– (*n* = 5)	0.00079 ± 0.4	0.011 ± 0.01	0.040 ± 0.01	0.024 ± 0.07	0.19 ± 0.4	0.90 ± 0.4
Labral instability						
+ (*n* = 18)	0.26 ± 0.2	0.0099 ± 0.007	0.010 ± 0.008	0.034 ± 0.03	0.49 ± 0.2	0.93 ± 0.5
– (*n* = 16)	0.13 ± 0.3	0.0022 ± 0.008	0.016 ± 0.008	0.10 ± 0.04	0.40 ± 0.2	0.63 ± 0.5
Chondral injury						
0 (*n* = 5)	0.013 ± 0.3	0.017 ± 0.01	0.044 ± 0.01	0.0080 ± 0.06	0.16 ± 0.5	0.055 ± 1.0
I (*n* = 16)	0.008 ± 0.2	0.017 ± 0.008	0.0086 ± 0.008	0.018 ± 0.03	0.41 ± 0.3	0.84 ± 0.5
II (*n* = 7)	0.0072 ± 0.2	0.022 ± 0.01	0.0080 ± 0.01	0.093 ± 0.05	0.62 ± 0.4	1.4 ± 0.8
III (*n* = 4)	0.53 ± 0.3	0.0013 ± 0.02	0.00051 ± 0.02	0.21 ± 0.07	0.56 ± 0.5	0.26 ± 1.1
IV (*n* = 2)	2.2 ± 0.5^*^	0.0069 ± 0.02	0.012 ± 0.02	0.23 ± 0.1	0.66 ± 0.7	1.0 ± 1.6
Synovitis						
2: focal (*n* = 16)	0.0038 ± 0.2	0.0053 ± 0.008	0.0035 ± 0.008	0.048 ± 0.04	0.44 ± 0.2	0.92 ± 0.5
3: diffuse (*n* = 18)	0.38 ± 0.2^*^	0.025 ± 0.007^*^	0.021 ± 0.008^*^	0.083 ± 0.04	0.46 ± 0.2^*^	0.68 ± 0.5

^*^ *P* < 0.05 was considered significant

### Correlation between hip pain, function of patients, and cytokine levels

We determined the correlations between the pain, loss of function experienced by patients, and synovial membrane cytokine levels (Table 4). VAS score during rest showed significant positive correlation with IL6 (*r* = 0.453, *p* = 0.0071), while VAS score on walking showed a significant positive correlation with TNFα (*r* = 0.465, *p* = 0.0056) and ADAMTS4 (*r* = 0.508, *p* = 0.0022). mHHS pain score showed a significant negative correlation with TNFα (*r* = −0.472, *p* = 0.0049), IL6 (*r* = −0.455, *p* = 0.0068), and ADAMTS4 (*r* = −0.349, *p* = 0.043). The mHHS total score showed a significant negative correlation with TNFα (*r* = −0.379, *p* = 0.04) and IL6 (*r* = −0.582, *p* = 0.0003).

**Table 4:** Correlations between hip pain, function, and cytokine levels

	VAS score at rest	VAS score on walking	mHHS	mHHS pain score
TNFα	*R* = 0.145 *P* = 0.41	*R* = 0.465 *P* = 0.0056^*^	*R* = −0.379 *P* = 0.04^*^	*R* = −0.472 *P* = 0.0049^*^
IL1β	*R* = −0.208 *P* = 0.24	*R* = −0.216 *P* = 0.22	*R* = −0.194 *P* = 0.27	*R* = −0.00617 *P* = 0.972
IL6	*R* = 0.453 *P* = 0.0071^*^	*R* = 0.292 *P* = 0.094	*R* = −0.582 *P* = 0.0003^*^	*R* = −0.455 *P* = 0.0068^*^
ADAMTS4	*R* = −0.00697 *P* = 0.97	*R* = 0.508 *P* = 0.0022^*^	*R* = −0.224 *P* = 0.2	*R* = −0.349 *P* = 0.043^*^
MMP1	*R* = −0.128 *P* = 0.47	*R* = 0.0150 *P* = 0.62	*R* = −0.167 *P* = 0.34	*R* = −0.124 *P* = 0.49
MMP3	*R* = −0.146 *P* = 0.38	*R* = 0.0211 *P* = 0.91	*R* = −0.165 *P* = 0.35	*R* = −0.107 *P* = 0.55

^*^ *P* < 0.05 was considered significant

## Discussion

In the current study, we examined the relationships between synovial cytokine levels, hip pain, and pathological findings during hip arthroscopy. We found pain tended to be more severe in patients with a worse grade of chondral injury or synovitis than in those with labral tear or instability. The expression of TNFα, IL1β, IL6, and MMP1 mRNA was significantly correlated with the severity of synovitis. We investigated the correlations between clinical pain scores and expression of IL6, TNFα, and ADAMTS4.

In knee OA, synovitis is recognized as a secondary phenomenon related to the cartilage degeneration induced by the release of cytokines or matrix proteases from articular cartilage into the synovial fluid, inducing knee pain (Higuchi et al. 2006; Smith et al. 1997; Inoue et al. 2011; Sellam and Berenbaum 2010). In the early stage of knee OA, the severity of synovitis is related to the pathogenesis of OA (Smith et al. 1997). Our results are consistent with these findings, and in patients with an indication for arthroscopy of the hip joint, chondral injury and severity of synovitis are important variables associated with hip joint pain.

Expression of TNFα, IL1β, IL6, and MMP1 mRNA was significantly higher in patients with diffuse synovitis, compared with patients with focal synovitis. Additionally, TNFα showed a tendency to be more highly expressed in patients with severe chondral injury. In knee OA, Smith et al. reported that the expression of TNFα mRNA in synovial membranes was positively correlated to the degree of articular cartilage degeneration and severity of synovitis (Smith et al. 1997). In hip joints, Abe et al. found significantly higher levels of TNFα, IL1β, and IL6 in the joint fluid in patients with severe OA compared with early OA (Abe et al. 2014). In patients with hip OA not seen radiographically, who were treated by arthroscopic surgery, we found there was significant positive correlation between TNFα and the severity of chondral injury, but we did not find significant correlations between IL1β or IL6 and the severity of injury. It remains unclear why the expression of IL1β, and IL6 mRNA was low in the present study, but in knee OA, an increased concentration of IL-6 was reported to correlate positively with the intensity of radiographic knee OA (Livshits et al. 2009). These cytokines might be correlated with a progressive stage of hip OA, and not an early stage, for which hip arthroscopy is indicated.

MMPs are important mediators in OA, and are in turn mediated by cytokines such as TNFα and IL1β, which are produced by synovial macrophages (Bondeson et al. 2006). For example, MMP1 is capable of cleaving collagen type II, and MMP3 is active against other components of the extracellular matrix (Klatt et al. 2009). ADAMTS4 is also an important inflammatory mediator in OA, and has degradative effects on the extracellular matrix (Abbaszade et al. 1999). In the present study, the level of MMP1 was significantly higher in the synovial membrane of patients with diffuse synovitis, than in those with focal synovitis, suggesting that severe synovitis can progress to catabolic chondral degeneration via MMP1, which is mediated by TNFα and IL1β.

TNFα sensitizes nociceptive neurons via the induction of a proinflammatory cytokine cascade involving IL-6, inducing inflammatory pain (Woolf et al. 1997; Sorkin et al. 1997). We found a positive correlation between IL6 and VAS score during rest, and TNFα, ADAMTS4 and VAS during walking. TNFα and IL6 also showed significant positive correlations with the mHHS pain score. These results suggest that TNFα and IL6 are involved in the production of pain, possibly depending on the severity of their synovitis, in patients indicated for arthroscopic hip surgery.

We did not find any significant correlation of labral tears or instability with either the severity of pain or synovial membrane cytokine levels in the present study. However, because of the small sample size used, the study may have lacked sufficient power to detect small differences in pain. Our results suggest that the severity of chondral injury and following synovitis, which reflects the severity of inflammation, might have more influence on pain severity than labral pathology in patients meeting the criteria for inclusion. Furthermore, our results suggest that labral tearing might not affect the severity of synovitis. In knee joints, asymptomatic meniscal tears are seen generally, and whether or not meniscal tears are symptomatic depends on the existence of local synovitis (Hunter et al. 2011; Guermazi et al. 2011). If labral tears in the hip do not essentially induce synovitis, then other factors might be necessary to produce pain in the hip joint. Management of synovitis and chondral injury during hip arthroscopic surgery might have a greater effect on the clinical outcomes, especially pain intensity, than management of labral tears.

Limitations of this study include its small sample size and lack of controls, for example patient participants with another hip diagnosis, or healthy volunteer participants without hip pain. The inability to find a significant correlation between pain intensity and cytokine levels may have been because the study lacked sufficient power to detect small differences in pain intensity as a result of the small sample size. A comparable study using a larger sample size is warranted to confirm our findings and elucidate the pathomechanism of hip joint pain in more detail.

## Conclusions

Severity of synovitis and chondral injury are considered to be more important pathologies for the origin of hip pain than labral tears or instability. Inflammatory cytokines, especially TNFα and IL6, might play important roles in the pathogenesis of pain in patients indicated for hip arthroscopy.

## Abbreviations

ADAMTS: A disintegrin and metalloproteinase with thrombospondin motifs
AP: Antero-posterior
CE: Centre-edge
FAI: Femoroacetabular impingement
IL: Interleukin
mHHS: modified harris hip score
MMP: Matrix metalloproteinase
MRI: Magnetic resonance imaging
OA: Osteoarthritis
TNF: Tumor necrosis factor
VAS: Visual analog scale
